# Summer habitat use and activity patterns of wild boar *Sus scrofa* in rangelands of central Argentina

**DOI:** 10.1371/journal.pone.0206513

**Published:** 2018-10-24

**Authors:** Nicolás Caruso, Alejandro E. J. Valenzuela, Christopher L. Burdett, Estela M. Luengos Vidal, Diego Birochio, Emma B. Casanave

**Affiliations:** 1 Grupo de Ecología Comportamental de Mamíferos (GECM), Instituto de Ciencias Biológicas y Biomédicas del Sur (INBIOSUR-CONICET), Departamento de Biología, Bioquímica y Farmacia, Universidad Nacional del Sur, Bahía Blanca, Buenos Aires, Argentina; 2 Instituto de Ciencias Polares, Ambiente y Recursos Naturales, Universidad Nacional de Tierra del Fuego y CONICET, Ushuaia, Tierra del Fuego, Argentina; 3 Department of Biology, Colorado State University, Fort Collins, Colorado, United States of America; 4 Escuela de Producción, Tecnología y Ambiente, Universidad Nacional de Río Negro, Sede Atlántica, Centro de Investigación y Transferencia Río Negro, Viedma, Río Negro, Argentina; Universitat Autonoma de Barcelona, SPAIN

## Abstract

Biological invasions are one of the main components of human-caused global change and their negative impact on invaded ecosystems have long been recognized. Invasive mammals, in particular, can threaten native biodiversity and cause economic impacts in the region where they are introduced, often through a wide range of conflicts with humans. Although the wild boar, *Sus scrofa*, is considered by the IUCN as one of the 100 invasive species most damaging to biodiversity in the world, in Argentina there have only been a few studies focused on its ecology with most of them conducted in protected areas. In this study, we evaluated the effect of several factors related with human disturbance, landscape composition, degree of fragmentation and the presence of a potential competitor and a predator on the habitat use of wild boar using data from camera traps and site-occupancy modeling. Additionally, we described the daily activity pattern of the species and we studied the level of overlap with both a potential competitor and a predator. The sampling effort totaled 7,054 camera trap days. Farm density, proportion of shrubland and proportion of grassland with bushes were the detection variables included in the most supported model whereas proportion of grassland and capture rate of the Pampas fox *Lycalopex gymnocercus* were the occupancy variables included in the most supported model. However, the proportion of grassland was the only variable that showed statistically significant support in the averaged model, indicating that habitat use of wild boar in this area was significantly negatively affected by the level of grass cover. Wild boars were mostly nocturnal, with more activity between 21:00 and 3:00 and a peak around midnight. Wild boars showed a high level of overlap with the activity pattern of the Pampas fox and a low overlap with the activity pattern of the puma *Puma concolor*. Despite wild boar being introduced in Argentina a few decades ago, this study is the first landscape-scale research carried out in an agricultural landscape in Argentina and the first one based on camera-trapping data. Our study contributes valuable information that could be used to design strategies to reduce wild boar population or to minimize the damage caused by this invasive species in Argentina.

## Introduction

Biological invasions constitute one of the main components of human-caused global change and their negative impact on the invaded ecosystem have long been recognized [1]. Exotic vertebrates have been introduced to almost all parts of the globe for thousands of years for different reasons, both purposeful and accidental [2]. Invasive mammals, in particular, can threaten native biodiversity [3] by various mechanisms such as reducing genetic variation of native species through hybridization, predation, competition, parasite transmission, and spread of diseases as well as modifying food webs, habitats and ecosystem functioning [4–7]. Furthermore, exotic mammal species can cause economic impacts in the region where they are introduced [8] and thus create a wide range of conflicts with humans [9]. Disease transmission represents a major conflict with humans in areas where livestock activities are important and contact between domestic and invasive species, both direct and indirect, are likely to happen [2].

The wild boar, *Sus scrofa*, is considered by the IUCN as one of the 100 invasive species most damaging to biodiversity in the world [10]. In non-native habitats, the species may act as an ecosystem engineer because it has strong negative effects on the superficial soil layers due to its rooting behavior. This behavior causes the mix of soil horizons and the alteration of the nutrient retention rate, which also enhance the erosion process, alters plant succession, favors exotic plant species invasion and may affect wildlife communities and complicated threatened and endangered species [11–14]. Furthermore, the wild boar has been mentioned as a potential predator of livestock, specially fawns, lambs and goat offspring [15–17]. On the other hand, several authors have also reported positive effects of wild boar introduction. In areas where hunting is an important source of economic income, wild boar may release native wildlife from over-harvesting acting as a replacement hunting target [18] Furthermore, in some areas this species might provide additional prey for native mammals [17].

Originally native to Eurasia, the species is currently present in all continents except Antarctica [19] and occupies a wide variety of natural habitats, from semi-desert to tropical rain forests, temperate woodlands, grasslands and reed jungles, as well as anthropogenic habitats, often foraging on agricultural land [20]. Although it prefers broadleaved forests in its native range, wild boar also occurs be found in more open habitats such as steppe, Mediterranean shrubland, and farmland provided there is water and tree cover nearby [21–23]. The capacity of wild boars to colonize different habitats and become an invasive species is due not only to their general habitat requirements, but also its biological characteristics, like the high reproduction rate, omnivorous diet, and behavioral plasticity [24–26]. Their ecological plasticity together with the growing urbanization process have allowed wild boars to colonize urban and periurban areas, increasing the risks associated with its presence and becoming a big concern for the government and managers [27].

In Argentina, the species was introduced during the early 20^th^ century for hunting purposes, but some individuals escaped from captivity and the species spread throughout a large part of the country [21,22]. Most studies on wild boars in Argentina have focused on abundance estimation [17,28], population occupancy tendencies [23], the impact of the species on soil and native flora [29,30], or its contribution to the establishment of invasive plants [31]. Fewer studies have focused on habitat use, with most of these being conducted in protected areas [32]. In the south of Argentina, wild boars tend to select low-altitudinal forest of *Nothofagus* spp. or mixed forest of *Araucaria*-*Nothofagus* spp. [23,33], and it has been proposed that the precipitation gradient in Patagonia may affect this pattern of habitat use, creating seasonal movements towards lower altitudes during winter [23]. Similarly, in arid areas of central-west Argentina, the species can change its habitat use between the dry and the wet season to maximize intake of high-quality food and to minimize exposure to high temperatures [34].

Knowing the habitat use and the activity patterns of an invasive species is vital to understanding their ecology and invasive potential, as it gives information about the adjustments that the species must make to fulfill for their normal activities of food gathering, mating, and caring for young [35]. Even though there are no previous studies of the activity pattern of wild boars in Argentina, studies elsewhere suggest important variations as well as great adaptability to external factors [25,36–39]. These changes in the activity pattern could be influenced by seasonality, as the climatic condition is an important factor in the availability of food and refuge [40,41], and by anthropogenic disturbances such as hunting [37]. Although interspecific competition and depredation can influence the distribution of wild boar [42,43], the species can also spatially and temporal reduce the niche overlap with potential competitors [44,45]. It therefore remains unclear how interspecific interactions influence wild boars, but it has been proposed that understanding its interspecific interactions with native species, despite being challenging, is necessary to understanding its distribution, particularly at local spatial scales [46]. Additionally, the information about the interactions with native carnivores is very limited [18].

In the present study, we evaluated landscape-level habitat use and daily activity pattern of wild boar in a region of the Argentine Espinal that has been modified by human activities. We used camera traps and site-occupancy modeling to study the effect of several factors related with human disturbance, landscape composition, degree of fragmentation, and intespecific interactions (i.e., the presence of one potential competitor, the Pampas fox, *Lycalopex gymnocercus*, and one predator, the puma, *Puma concolor*) on the habitat use of wild boar. We chose these species to study intespecific interactions because the puma is the top predator of the mammal community in our study area and has been shown that can prey on wild boars in the Espinal [47] whereas the Pampas fox, like the wild boar, is also a habitat and dietary generalist [48]. Additionally, we described the daily activity pattern of the species and we studied the level of overlap with its potential competitor and predator.

## Methods

### Study area

**Fig 1:**
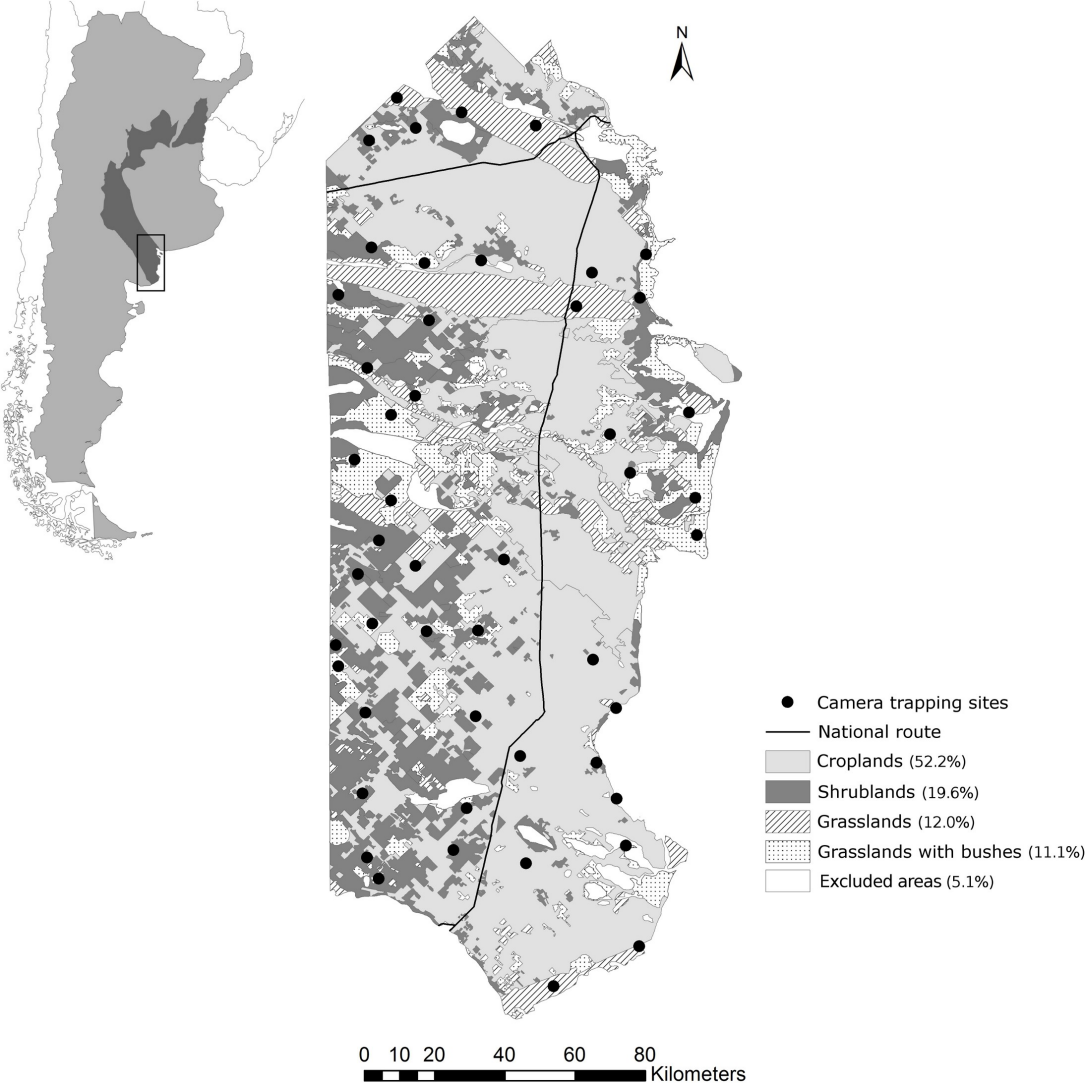
Study area. Map of the study area with the locations of camera trap sites. The dark area in the general map of Argentina represents the Espinal ecorregion. Modified from [52].

The study area belongs to the Argentine Espinal ecoregion and is characterized by a temperate, semiarid climate, where aridity increases towards the west and south [49–51]. The mean annual temperature is 15.3°C. The annual precipitation varies from 350 to 550 mm and occurs mainly during spring and autumn. The topography is mostly flat and natural vegetation is characterized by xerophytic deciduous woodlands, prairies dominated by grasslands, and prairies intermixed with extensive shrublands (henceforward, grassland with shrubs). This region has experienced a marked transformation during the last decades due to the increase of agriculture and ranching activities, which are the most important sources of income regionally [50]. These activities have fragmented the original landscape into a mosaic of croplands and pastures with residual patches of original vegetation, and thus altered the natural habitat. Fieldwork was conducted on private farms representing a total area of 27,300 km^2^ located in central Argentina and corresponding to the southernmost counties of the Buenos Aires province (Fig 1).

### Data collection

Camera trap surveys were conducted from January to March in 2011, 2012, and 2013 as a part of a broader study of mammal occurrence. To select the sampling sites, we used a geographic information system (GIS) to select 100 random points with a distance among them of at least 6 km (see [52,53] for sampling design details) (Fig 1). Then, we deployed cameras in 49 of those points (23 in 2011, 14 in 2012 and 12 in 2013; hereafter “sites”) because of logistical restrictions and adjustment of the survey design to the number of cameras available. Within each site, we used a set of Bushnell Trophy, Reconyx Rapidfire/Hyperfire and ScoutGuard SG550 cameras which were installed near trails or places with indirect evidences of the target species (feaces or foot tracks) and were baited using a piece of plaster embedded with either Bobcat urine extract or Bobcat gland extract. Each survey was conducted for 35 days (range 25–45) and all cameras were operational 24 hours per day. Cameras were checked every 5-7 days to replace the batteries and memory cards and to ensure their proper functioning. Total sampling effort was calculated as the sum of the effective days across all stations that each camera was functioning [54]. We considered photos separated by at least 30 minutes as independent events [38,55].

### Predictive variables

To evaluate if anthropogenic disturbance, landscape composition and degree of fragmentation affects habitat use, 12 variables related to these drivers were measured (Table 1). Additionally, we use the capture rate of the Pampas fox (*Lycalopex gymnocercus*) and puma (*Puma concolor*) as a covariate to assess the effect of the presence of potential competitors or predators. Further, to test the effect of different survey effort among sites, we incorporated the effective number of sampling days per site into our models. Spatial variables were calculated within an area of 1.5 km radius around each camera site using a vector land cover map provided by the National Institute of Agriculture Technology (INTA). Distance variables were calculated as the Euclidean distance from the center of each site to the closest landscape element of interest. “Farm density” was calculated as the number of farms within the buffer limits divided by buffer area, using a cadastral map of the study area provided by INTA. All variables were calculated using ArcGIS 10 [56] and Fragstats 4.11 [57].

**Table 1:** Predictive variables.

Variable (unit)	Description
Farm density (n° of farms/km^2^)	Number of properties per km^2^
Distance to settlements (km)	Euclidean distance in km from the site to the closest urban settlement
Distance to main route (km)	Euclidean distance in km from the site to the main route
Proportion of cropland	Proportion of the buffer area occupied by the category “cropland”
Proportion of shrubland	Proportion of the buffer area occupied by the category “shrubland”
Proportion of grassland	Proportion of the buffer area occupied by the category “grassland”
Proportion of grassland with bushes	Proportion of the buffer area occupied by the category “grassland with bushes”
Croplands’ edge density (km/km^2^)	Total length of the edges between “croplands” and the other categories divided by the buffer area
Shannon Diversity Index	Calculated on land cover categories within the buffer area
Capture rate for *Lycalopex gymnocercus*	Number of pictures of the species divided by the effective sampling effort per site
Capture rate for *Puma concolor*	Number of pictures of the species divided by the effective sampling effort per site
Effecting sampling effort	Calculated as the log of the number of effective sampling days per site

The predictive variables used to fit single-species single-season occupancy models for wild boar *Sus scrofa* in rangelands of central Argentina.

### Site occupancy models

Single-species single-season occupancy models were fitted to study the patterns of habitat use of wild boar [58,59]. Occupancy was defined as the proportion of sites used by the species and detection as the probability that wild boars were detected during a survey occasion (7 camera trapping days) at each site used by the species. The main assumption of this approach is that the occupancy status of the species remains unchanged during the sampling period (i.e., the closure assumption) [58]. Despite the short duration of our survey period, movements in and out of the study area may still occur and, assuming these movements are random, occupancy should be interpreted as habitat use rather than the proportion of area occupied by the species [60]. Detection histories combining the detection history of each of the 5 cameras within a site were constructed for each site to indicate if the species was detected or undetected. Detection status for a given site during a given survey occasion was set as a missing observation only if none of the 5 cameras within the site were functional.

To avoid bias due to multi collinearity, we examined Pearson correlation coefficients between pairs of the predictive variables prior to model building [61]. All predictive variables were z-transformed prior to analysis. To avoid fitting a large number of models relative to the size of our dataset, we use the procedure applied by [62] (see page 590) based on a stepwise model selection process first on the detection part (while keeping the occupancy parameter constant) and then on the occupancy part (while keeping the detection part as identified in the first step). Then the models were ranked using the Akaike Information Criterion corrected for small sample size (AIC_c_) [63]. As recommended by MacKenzie and Bailey [64], the model fit was assessed using the Goodness of Fit test (GoF) proposed by the authors and the mean dispersion parameter (*ĉ*) was estimated for global model using 1000 parametric bootstraps. In those cases, where the global model was found to have a poor fit (i.e., GoF *p* value < 0.05), we used *ĉ* to correct the AICc and used its quasi-likelihood version instead to rank the models [63]. We reported model-averaged predictions, parameter estimates, unconditional standard errors and 95% confidence intervals for model sets defined by ΔAICc < 2. The strength of evidence for each predictive variable was determined by evaluating the model averaged parameter estimates (i.e., the corresponding *β* for each variable in the model) with respect to zero using unconditional standard errors and 95% confidence intervals [63]. Models were fitted to the data using the packages “unmarked” [65] and “MuMIn” [66] in R version 3.4.3 [67].

### Daily activity patterns

Kernel-density estimation was used to describe temporal activity of wild boar. This method considers each photographic record as a random sample of an underlying continuous distribution, instead of grouping photographic records in blocks of predefined discrete time categories [68]. Following the suggestion of [59], we chose the kernel concentration parameter of k = 3 with smoothness parameter of c = 1 for these calculations. To investigate the level of overlap between the wild boar, Pampas fox and puma, we used the coefficients of overlap proposed by Ridout and Linkie [68] in pairwise comparisons. Those coefficients estimate the level of overlap between two activity distributions, ranging from 0 for no overlap, to 1 for identical distributions. As Ridout and Linkie [68] suggest, we applied the Δ_1_ estimator in those cases were the smallest number of photographic records was less than 50, and the Δ_4_ estimator when this number was higher than 50. We obtained confidence intervals for each estimator using a bootstrap procedure [69]. The analysis was performed using the package “overlap” [70] implemented in R version 3.4.3 [67].

## Results

The sampling effort totaled 7,054 camera trap days. Wild boars were detected at 29 of the 49 sites (59.2%) at least once during the sampling period in a total of 159 independent events with at least one individual.

Since distance to urban settlements was strongly correlated to distance to main road (*r* > 0.6), only distance to main road was used into the analysis. All other variables did not show any strong correlation among them (all *r* < 0.4).

**Fig 2:**
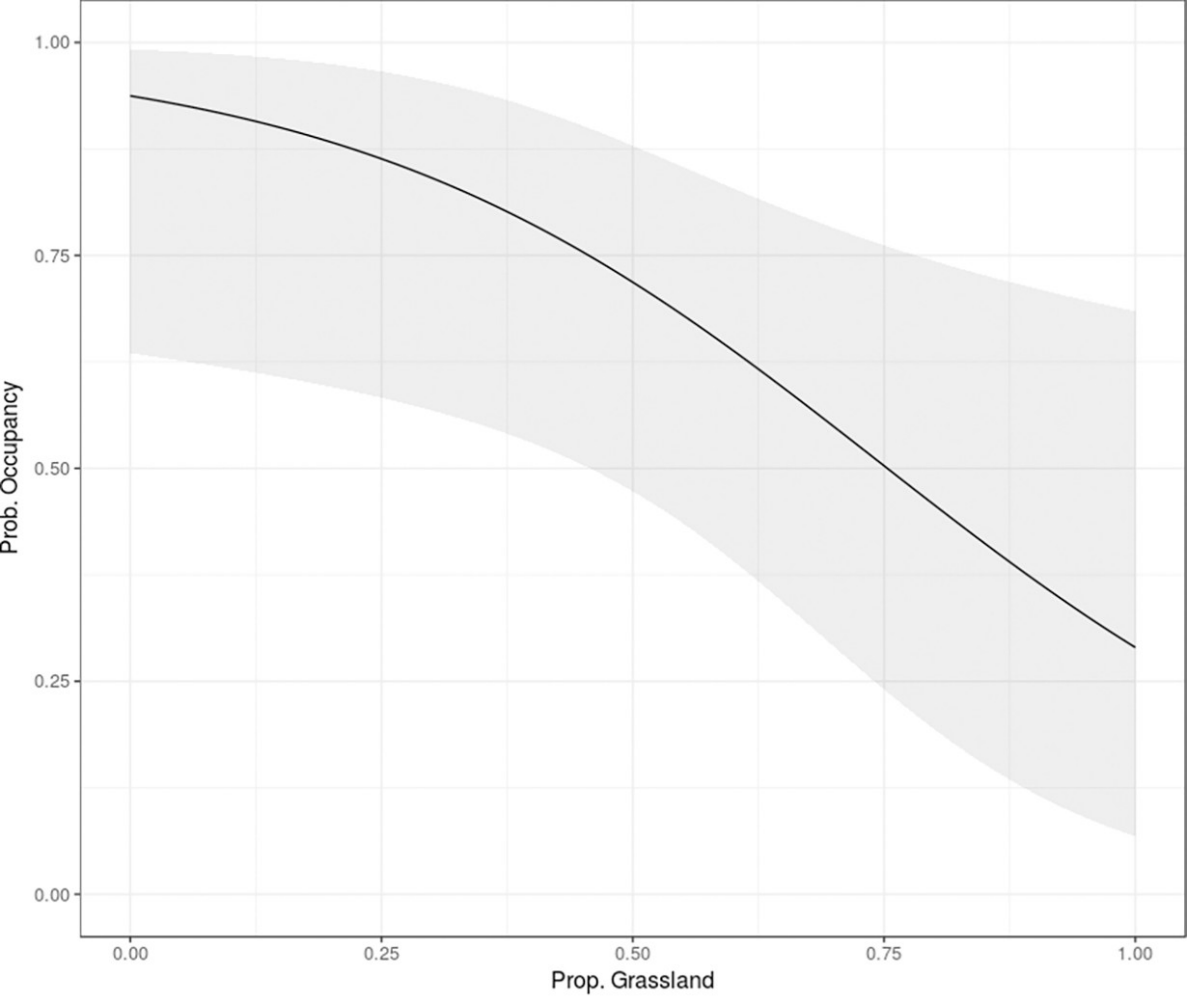
Site-occupancy. Estimated relationship between the site-occupancy probability and the proportion of grassland for wild boars *Sus scrofa* in central Argentina. The shaded area shows the 95% confidence interval.

Farm density, proportion of shrubland, and proportion of grassland with bushes were the detection variables included in the most supported model whereas proportion of grassland and capture rate of the Pampas fox were the occupancy variables included in the most supported model (Table 2 and S1 Table). In total, 32 models were fit using all possible combinations of those variables and no single model was clearly better, with 5 models having ΔAICc < 2 (Table 2 and S1 Table). Both proportion of grassland with bushes and proportion of shrubland showed a positive relationship with the detection parameter (*β*_*PGB*_ = 0.288, *β*_*PS*_ = 0.238), while the estimated *β* of farm density was negative (*β*_*FD*_ = -0.151). For the occupancy parameter, proportion of grassland showed a negative relationship (*β*_*PG*_ = -0.853) with the response variable, while the capture rate of *L*. *gymnocercus* showed a positive relationship (*β*_*CR_L*_ = 1.63). However, the proportion of grassland was the only variable that showed statistically significant support in the averaged model (95% CI_PG_ = -1.70, -0.02) suggesting that wild boar tends to less frequently use sites with a high proportion of herbaceous vegetation (Fig 2).

**Table 2:** List of the top-five single-species single-season occupancy models fit for wild boar *Sus scrofa* in rangelands of central Argentina and the average model.

**Model**	**ψ(Int)**	*p***(Int)**	**ψ(PG)**	**ψ(CR_L)**	*p***(FD)**	*p***(PS)**	*p*(PGB)	AICc	ΔAICc
ψ(PG+CR_L) *p*(PGB)	0.907	-0.307	-0.851	1.579	-	-	0.256	273.98	0.000
ψ(PG+CR_L) *p*(.)	0.832	-0.249	-0.834	1.525	-	-	-	274.11	0.130
ψ(PG+CR_L) *p*(PS+PGB)	1.086	-0.408	-0.890	1.823	-	0.238	0.363	275.22	1.239
ψ(PG+CR_L) *p*(FD)	0.936	-0.252	-0.881	1.754	-0.151	-	-	275.77	1.789
ψ(PG) *p*(PGB)	0.612	-0.324	-0.825	-	-	-	0.266	275.95	1.974
**Average model**	**0.885 (-0.234, 2.003)**	**-0.302 (-0.679, 0.075)**	**-0.853 (-1.691, -0.015)**	**1.445 (-0.775, 4.038)**	**-0.019 (-0.476, 0.173)**	**0.039 (-0.161, 0.636)**	**0.168 (-0.052, 0.627)**		

Ψ: occupancy parameter; *p*: detection parameter; df: degree of freedom; AICc: Akaike Information Criterion corrected for small sample size; W_i_: Akaike weight. PG: proportion of grassland, PGB: proportion of grassland with bushes, PS: proportion of shrubland, FD: farm density, CR_L: capture rate of Pampas fox *Lycalopex gymnocercus*.

**Fig 3:**
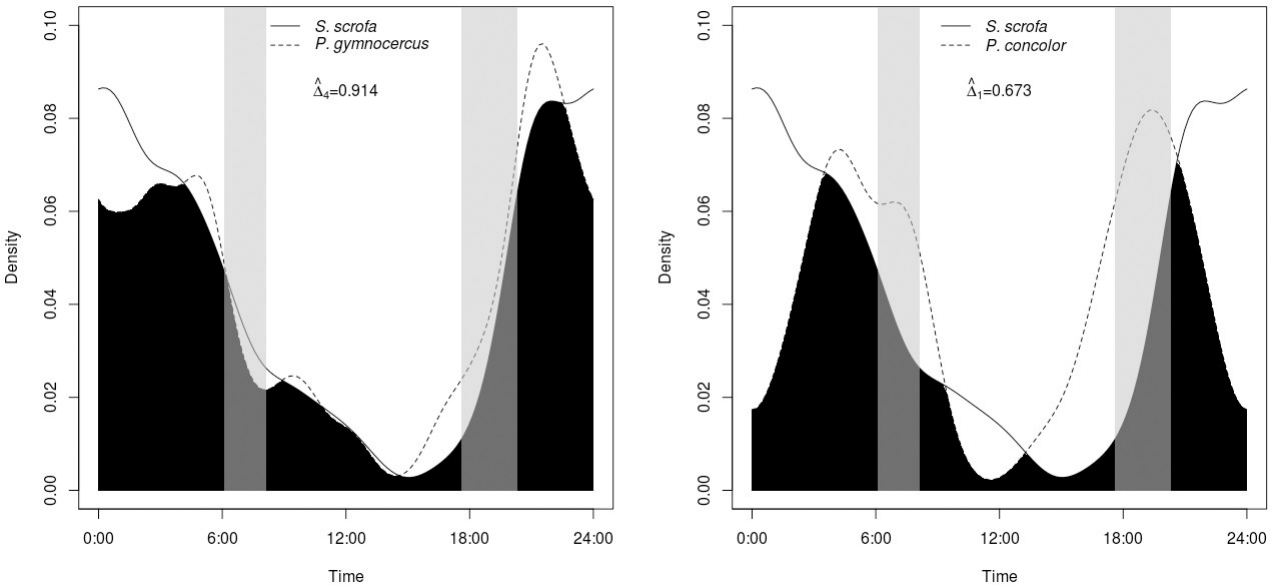
Activity patterns. Pairwise comparison of daily activity patterns of wild boar *Sus scrofa*, with a) Pampas fox *Lycalopex gymnocercus*, and b) the puma *Puma concolor* in central Argentina. Black area denotes overlap of activity. Grey columns represents sunrise and sunset.

Wild boars were mostly nocturnal, with more activity between 21:00 and 3:00 (76.7% of the independent events) with a peak around midnight (Fig 3). The highest rate of increase in activity was during dusk (approximately at 19:30, Fig 3). Few pictures were recorded during daytime (11.3% of the independent events), with most of those occuring during mornings hours. The activity pattern of the wild boar was very similar to the pattern of the Pampas fox (Δ_4_ = 0.914; CI = 0.88 – 0.99), while the coefficient of activity overlap between the wild boar and the puma was considerably lower (Δ_1_ = 0.673; CI = 0,52 – 0,80).

## Discussion

To our knowledge, previous ecological studies of the wild boar in Argentina have been conducted in protected areas, and this study is the first conducted in an agricultural landscape. Furthermore, this is the first camera-trap based data on habitat use and activity patterns of wild boars in Argentina. Even though the southern part of the Espinal was the place where the first individuals were released [22], there is little information available about the ecology of this species in the area, its interaction with native species and the impact upon the ecosystem [71].

Habitat use of wild boar in this area was significantly negatively affected by the proportion of grass cover. These results agreed with previous studies where the species is mainly described as a forest or forest-edge species and mostly occurred near natural habitats [34,72–74]. While wild boars regularly use forest and shrublands [75], several authors have suggested that the species is able to use open areas but requires trees or bush patches for shelter [72,73,76]. In our study area, grasslands are open habitats that provide little cover and likely do not offer abundant food resources relative to other habitats (i.e., grassland with bushes or shrubland). Both proportion of grassland with bushes and proportion of shrubland showed a positive relationship with the detection parameter, even when their support in the averaged model was not statistically significant, which suggests that wild boars are more detectable in those habitats. Detectability may be lower in open habitats than in closed habitats because in the latter there are trails created by livestock that are frequently used by wildlife. We usually installed cameras near these trails to increase the detection rate of the target species (see Methods). In contrast, in open areas there are no such a clear trails and animals can pass by close to the camera without being detected (e.g., walking behind the camera). However, we believe that the higher detection in more closed areas is a result of the higher use rate of that habitat by wild boars. Temperature and water availability are two additional and important factors affecting distribution and abundance of the wild boar [34,77,78]. Since this study was carried out mostly during summer when temperatures can be above 30°C and the water availability is at its lowest, seasonality could also explain the avoidance of grassland.

With the exception of proportion of grassland, most of the predictive variables included to construct the models describing habitat use did not show statistically significant support. This result may be related to the plasticity of wild boar habitat use, which has already been found elsewhere in Argentina [34]. The wild boar’s invasion and establishment success is a consequence of the species’ high capacity to adapt to different conditions, being both a habitat and dietary generalist [2]. Furthermore, the wild boar coexists with only one potential natural predator in our study area, the puma. The threatened conservation status and low abundance of the puma in this area [79], mainly due to persecution by farmers and habitat fragmentation [79], could have decreased this potential predator–prey interaction [24], reducing wild boars reducing wild boars behavioral need to use vegetation for defensive cover. Accordingly, we did not find any relationship between the capture rate of pumas and the probability of occupancy or detection of wild boars. However, the capture rate of the Pampas fox was positively related to the probability of occupancy of the wild boar, suggesting that both species use similar habitats. Although there is little information about interactions between the wild boar and other species, *L*. *gymnocercus* is also a generalist species able to adjust its habitat use and diet to resource availability [52,80]. Thus, dietary overlap could explain the positive habitat association between the Pampas fox and wild boar, as has been suggested for other foxes [2,81,82].

Wild boars exhibited a crepuscular and nocturnal activity pattern that peaked around midnight. These results agree with the previous knowledge about its general daily activity patterns [75]. Even though the species is mainly nocturnal, it may have a certain level of flexibility and become active during daytime, especially in undisturbed areas or in areas with nocturnal hunting [37,83]. In our study area wild boars are highly persecuted by farmers because the species is considered a pest (mostly due to crop damage) and also for personal consumption (Caruso *et al*. unpublished data). Because ranchers hunt during night in this area we would have expected a shift in the activity pattern toward daytime. However, it is more likely that, since the study was carried out during summer, wild boars preferred being active during nighttime to avoid high daytime temperatures. Several publications have documented the seasonal change of activity patterns of wild boar resulting from thermoregulation [83–85]. We would be to replicate our research during the winter to better understand the different trade-offs involved with the influence of human activity and temperature on wild boar activity patterns.

Similar to our findings for habitat use, the activity pattern of wild boar showed a high coefficient of overlap with the activity pattern of the Pampas fox; however, the level of overlap with the activity pattern of the puma was relatively low. Pumas showed two daily moments of higher activity, one during the sunrise and the hours immediately prior, which matches when the wild boar decreased its level of activity, and another during the sunset before the peak of activity for wild boars. The puma is the only potential predator of wild boars in our study area [18,86,87], which may explain the low overlap in the activity patterns of these two species. [18,86,87].

In summary, we found that the wild boar showed little use of grasslands when other type of habitats is available. The species is more active during the night and presents a spatio-temporal overlap with the Pampas fox and a temporal segregation with the puma. Despite wild boar being introduced in Argentina a few decades ago, information regarding its general ecology and the impact that its populations have on the Espinal ecoregion remains scarce. Our study contributes valuable information relative to habitat use, activity patterns, and interactions with native wildlife in a highly anthropogenic area of Central Argentina, where agriculture and ranching are a dominant land use. This information could be used to design strategies to reduce wild-boar populations or minimize the damage caused by this invasive species in Argentina. Knowledge of habitat use and activity patterns of an exotic invasive species provide critical information to help decision makers to plan management strategies [88,89]. In particular, our results could be used to decide where and when control actions should be taken in order to reduce wild-boar populations or minimize the damage caused by this invasive species in Argentina. From this information, seasonal potential distribution maps of wild boars could be created helping to understand the spatial patterns of the species, which serve to identify the sites where to carry out the control, or avoid spatial interaction with livestock in order to reduce disease transmission (e.g., install portable drinking troughs in places not used by wild boars, i.e. in open areas or near them, to prevent wild boar sharing drinking places with livestock). Moreover, habitat use information might be used to improve vegetation management practices to reduce economic damage to farms and ranches. In the order hand, temporal patterns found in this study could be also used in the planning of the timing of control actions to manage wild-boar population in the area. We recommend long-term monitoring of wild-boar population in human-dominated landscapes which would help to understand not only seasonal changes in habitat use and activity patterns, but also to test the effectiveness of different management strategies.

## Supporting information

S1 Table List of the 32 single-species single-season occupancy models fit for wild boar *Sus scrofa* in rangelands of central Argentina. Ψ: occupancy parameter; *p*: detection parameter; df: degree of freedom; AICc: Akaike Information Criterion corrected for small sample size; W_i_: Akaike weight. PG: proportion of grassland, PGB: proportion of grassland with bushes, PS: proportion of shrubland, FD: farm density, CR_L: capture rate of Pampas fox *Lycalopex gymnocercus*.
(PDF)

S2 Table Raw data. For each site the table shows the capture history for wild boar *S*. *scrofa* and the value of each predictor using to fit single-species single-season occupancy models. O1-O6: occasions, FD: farm density, DS: distance to settlements, PC: proportion of cropland, PS: proportion of shrubland, PG: proportion of grassland, PGB: proportion of grassland with bushes, CED: cropland edge density, SDI: Shannon Diversity Index, CR_P: capture rate of puma *Puma concolor*, CR_L: capture rate of Pampas fox *Lycalopex gymnocercus*, DUR: effective sampling effort.
(PDF)
