# Correction: Summer habitat use and activity patterns of wild boar *Sus scrofa* in rangelands of central Argentina

**DOI:** 10.1371/journal.pone.0207722

**Published:** 2018-11-15

**Authors:** Nicolás Caruso, Alejandro E. J. Valenzuela, Christopher L. Burdett, Estela M. Luengos Vidal, Diego Birochio, Emma B. Casanave

The following information is missing from the Funding section: This study was supported by Proyecto de Investigación Científica y Tecnológica—Agencia Nacional de Promoción Científica y Tecnológica (PICT-2015-2283) - http://www.agencia.mincyt.gob.ar/.
