# Supplementary material for: Summer habitat use and activity patterns of wild boar Sus scrofa in rangelands of central Argentina
Source: PLoS One. 2018 Oct 24;13(10):e0206513. doi: 10.1371/journal.pone.0206513 (PMC6200264; doi:10.1371/journal.pone.0206513)
Supplement: S1 Table — Ψ: occupancy parameter; p: detection parameter; df: degree of freedom; AICc: Akaike Information Criterion corrected for small sample size; Wi: Akaike weight. PG: proportion of grassland, PGB: proportion of grassland with bushes, PS: proportion of shrubland, FD: farm density, CR_L: capture rate of Pampas fox Lycalopex gymnocercus. (PDF) [file pone.0206513.s001.pdf]

Model	ψ(Int)	*p*(Int)	ψ(PP)	ψ(CR_L)	p(FD)	p(PS)	p(PGB)	df	logLik	AICc	ΔAICc	Wi
ψ(PG+CR_L) p(PGB)	0.907	-0.307	-0.851	1.579	-	-	0.256	5	-131.29	273.98	0.000	0.148
ψ(PG+CR_L) p(.)	0.832	-0.249	-0.834	1.525	-	-	-	4	-132.60	274.11	0.130	0.138
ψ(PG+CR_L) p(PS+PGB)	1.086	-0.408	-0.890	1.823	-	0.238	0.363	6	-130.61	275.22	1.239	0.079
ψ(PG+CR_L) p(FD)	0.936	-0.252	-0.881	1.754	-0.151	-	-	5	-132.19	275.77	1.789	0.060
ψ(PG) p(PGB)	0.612	-0.324	-0.825	-	-	-	0.266	4	-133.52	275.95	1.974	0.055
ψ(PG) p(.)	0.547	-0.262	-0.814	-	-	-	-	3	-134.91	276.35	2.374	0.045
ψ(PG+CR_L) p(FD+PGB)	0.879	-0.318	-0.832	1.488	0.075	-	0.305	6	-131.24	276.48	2.505	0.042
ψ(PG+CR_L) p(PS)	0.848	-0.262	-0.838	1.550	-	0.041	-	5	-132.57	276.54	2.560	0.041
ψ(CR_L) p(PGB)	0.784	-0.309	-	1.233	-	-	0.261	4	-133.94	276.79	2.816	0.036
ψ(PG) p(PS+PGB)	0.722	-0.432	-0.831	-	-	0.243	0.377	5	-132.82	277.04	3.062	0.032
ψ(PG+CR_L) p(FD+PS+PGB)	1.064	-0.498	-0.812	1.521	0.255	0.343	0.580	7	-130.16	277.05	3.073	0.032
ψ(CR_L) p(.)	0.720	-0.246	-	1.214	-	-	-	3	-135.29	277.12	3.146	0.031
ψ(CR_L) p(FD+PS+PGB)	1.178	-0.622	-	1.145	0.424	0.475	0.772	6	-131.69	277.38	3.401	0.027
ψ(.) p(FD+PS+PGB)	1.186	-0.698	-	-	0.494	0.528	0.858	5	-132.99	277.38	3.404	0.027
ψ(CR_L) p(PS+PGB)	0.981	-0.446	-	1.384	-	0.281	0.398	5	-133.07	277.54	3.568	0.025
ψ(PG) p(FD+PS+PGB)	0.906	-0.578	-0.737	-	0.359	0.407	0.695	6	-131.89	277.79	3.808	0.022
ψ(PG) p(FD+PGB)	0.633	-0.351	-0.809	-	0.132	-	0.355	5	-133.35	278.10	4.120	0.019
ψ(PG) p(FD)	0.559	-0.261	-0.829	-	-0.120	-	-	4	-134.64	278.18	4.204	0.018
ψ(PG+CR_L) p(FD+PS)	0.965	-0.268	-0.890	1.804	-0.155	0.053	-	6	-132.14	278.28	4.300	0.017
ψ(PG) p(PS)	0.554	-0.274	-0.814	-	-	0.038	-	4	-134.88	278.68	4.702	0.014
ψ(CR_L) p(FD+PGB)	0.799	-0.346	-	1.156	0.161	-	0.371	5	-133.71	278.82	4.840	0.013
ψ(.) p(PGB)	0.622	-0.334	-	-	-	-	0.274	3	-136.15	278.83	4.855	0.013
ψ(CR_L) p(FD)	0.744	-0.242	-	1.277	-0.115	-	-	4	-135.05	279.01	5.035	0.012
ψ() p(PS+PGB)	0.813	-0.492	-	-	-	0.308	0.427	4	-135.14	279.18	5.207	0.011
ψ(CR_L) p(PS)	0.733	-0.261	-	1.225	-	0.046	-	4	-135.26	279.43	5.451	0.010
ψ(.) *p*(.)	0.550	-0.265	-	-	-	-	-	2	-137.61	279.47	5.496	0.009
ψ(FD) p(PGB)	0.704	-0.397	-	-	0.224	-	0.432	4	-135.69	280.29	6.311	0.006
ψ(PG) p(FD+PS)	0.569	-0.275	-0.831	-	-0.123	0.045	-	5	-134.60	280.60	6.625	0.005
ψ(CR_L) p(FD+PS)	0.761	-0.259	-	1.295	-0.117	0.051	-	5	-135.01	281.41	7.438	0.004
ψ(.) p(FD)	0.549	-0.258	-	-	-0.093	-	-	3	-137.44	281.42	7.443	0.004
ψ(.) p(PS)	0.563	-0.282	-	-	-	0.053	-	3	-137.56	281.66	7.678	0.003
ψ(.) p(FD+PS)	0.562	-0.276	-	-	-0.094	0.053	-	4	-137.40	283.71	9.728	0.001
