# Supplementary material for: Summer habitat use and activity patterns of wild boar Sus scrofa in rangelands of central Argentina
Source: PLoS One. 2018 Oct 24;13(10):e0206513. doi: 10.1371/journal.pone.0206513 (PMC6200264; doi:10.1371/journal.pone.0206513)
Supplement: S2 Table — For each site the table shows the capture history for wild boar S. scrofa and the value of each predictor using to fit single-species single-season occupancy models. O1-O6: occasions, FD: farm density, DS: distance to settlements, PC: proportion of cropland, PS: proportion of shrubland, PG: proportion of grassland, PGB: proportion of grassland with bushes, CED: cropland edge density, SDI: Shannon Diversity Index, CR_P: capture rate of puma Puma concolor, CR_L: capture rate of Pampas fox Lycalopex gymnocercus, DUR: effective sampling effort. (PDF) [file pone.0206513.s002.pdf]

SITE	O1	O2	O3	O4	O5	O6	FD	DS	PC	PS	PG	PGB	CED	SDI	CR_P	CR_L	DUR
S100P	0	0	0	0	NA	NA	0.28	30.73	0.26	0.70	0.04	0.00	0.67	0.73	0.00	0.02	121.35
S10V	1	0	1	1	0	0	0.38	33.82	0.33	0.10	0.09	0.28	0.90	1.53	0.00	0.09	172.02
S11V	1	0	0	0	0	0	0.21	41.18	0.00	0.00	0.15	0.00	0.12	0.44	0.05	0.48	171.33
S12P	0	0	0	1	NA	NA	0.24	24.48	1.00	0.00	0.00	0.00	0.91	0.00	0.01	0.02	85.20
S12V	0	0	0	0	0	0	0.20	24.27	0.37	0.05	0.27	0.00	0.97	0.98	0.00	0.02	206.53
S13V	0	1	1	0	1	0	0.16	20.84	0.23	0.19	0.00	0.58	0.66	1.03	0.01	0.20	210.07
S14V	0	1	1	0	0	NA	0.35	42.57	0.00	0.42	0.17	0.41	0.00	1.17	0.00	0.02	99.07
S1P	0	0	0	0	NA	NA	0.41	17.44	0.49	0.04	0.23	0.00	0.85	0.29	0.00	0.00	131.71
S1V	0	0	0	0	0	0	0.32	22.84	0.08	0.00	0.00	0.92	0.63	0.55	0.00	0.04	142.22
S24P	1	0	0	0	NA	NA	0.17	54.18	0.24	0.00	0.00	0.76	0.81	1.17	0.01	0.10	136.11
S28P	0	1	0	0	0	0	0.28	25.92	0.24	0.55	0.00	0.21	0.84	0.48	0.00	0.04	205.15
S29P	1	0	0	0	0	NA	0.30	24.44	0.00	0.82	0.18	0.00	0.00	0.84	0.00	0.01	96.04
S2V	1	0	0	0	1	0	0.31	37.38	0.50	0.49	0.01	0.00	1.21	0.65	0.00	0.02	195.31
S31P	0	0	0	0	NA	NA	0.22	19.06	0.36	0.64	0.00	0.00	0.87	0.94	0.00	0.01	137.14
S35P	0	0	0	0	NA	NA	0.33	12.48	0.13	0.00	0.00	0.29	0.44	0.95	0.00	0.00	123.83
S36P	0	0	0	0	NA	NA	0.32	11.23	0.59	0.15	0.00	0.00	0.87	1.17	0.00	0.00	100.98
S3P	1	1	1	0	NA	NA	0.29	9.76	0.35	0.32	0.00	0.02	0.83	1.12	0.00	0.00	132.21
S3V	1	1	1	1	1	0	0.26	47.77	0.16	0.58	0.17	0.00	0.56	0.73	0.00	0.03	151.44
S41P	0	0	0	0	NA	NA	0.45	4.28	1.00	0.00	0.00	0.00	0.90	0.00	0.00	0.07	131.56
S43P	0	0	0	0	0	NA	0.15	45.40	0.00	0.00	0.35	0.65	0.00	0.65	0.03	0.03	144.62
S44P	0	1	1	1	0	NA	0.22	56.81	0.21	0.79	0.00	0.00	0.95	0.51	0.01	0.02	111.92
S4V	1	1	1	1	1	0	0.46	17.22	0.29	0.71	0.00	0.00	0.63	0.61	0.01	0.04	193.85
S53P	0	0	0	0	0	NA	0.23	56.28	0.05	0.95	0.00	0.00	0.27	0.62	0.03	0.05	132.03
S55P	0	0	1	0	0	NA	0.20	51.66	0.03	0.97	0.00	0.00	0.21	0.12	0.01	0.02	168.21
S56P	1	0	1	1	NA	NA	0.22	23.62	0.23	0.46	0.00	0.31	0.56	1.07	0.00	0.04	124.65
S5V	1	1	1	1	1	0	0.26	27.07	0.08	0.83	0.00	0.08	0.44	0.57	0.02	0.55	144.55
S61P	0	0	0	0	1	0	0.11	52.96	0.00	0.12	0.00	0.88	0.00	0.37	0.01	0.03	181.66
S62P	1	0	0	1	NA	NA	0.38	44.69	0.37	0.63	0.00	0.00	1.15	0.66	0.00	0.18	125.29
S66P	0	0	0	0	NA	NA	0.22	63.89	0.01	0.62	0.37	0.00	0.17	1.09	0.00	0.06	125.00
S68P	1	0	0	0	0	NA	0.18	38.52	0.37	0.60	0.03	0.00	0.89	1.20	0.00	0.02	125.63
S6V	0	0	0	1	0	0	0.52	15.08	0.73	0.27	0.00	0.00	1.15	0.58	0.00	0.14	180.46
S71P	0	0	0	0	NA	NA	0.12	34.06	0.00	0.00	1.00	0.00	0.00	0.00	0.00	0.00	103.86
S7P	0	0	0	0	0	0	0.12	39.67	0.54	0.22	0.23	0.00	1.20	1.01	0.00	0.06	187.27
S7V	0	0	0	1	1	0	0.36	28.29	0.22	0.78	0.00	0.00	1.06	1.00	0.00	0.04	171.28
S8V	0	1	0	0	0	1	0.64	7.78	0.00	0.00	1.00	0.00	0.00	0.00	0.00	0.11	144.47
S99P	1	1	0	0	NA	NA	0.20	61.30	0.21	0.11	0.02	0.65	0.45	0.93	0.00	0.02	121.66
S9V	0	0	1	1	0	0	0.20	23.16	0.07	0.67	0.00	0.25	0.37	0.84	0.00	0.08	143.94
SA	1	1	0	1	1	1	0.04	43.47	0.07	0.00	0.00	0.93	0.21	0.25	0.04	0.24	161.27
SB	0	0	0	0	0	0	0.23	17.09	0.85	0.07	0.00	0.08	1.11	0.52	0.00	0.05	90.91
SC	0	0	0	0	0	0	0.48	13.75	0.93	0.07	0.00	0.00	1.22	0.25	0.01	0.01	164.98
SD	1	1	1	1	1	NA	0.05	42.16	0.02	0.00	0.00	0.97	0.13	0.16	0.01	0.07	149.31
SE	1	0	0	0	0	NA	0.25	31.41	0.57	0.00	0.16	0.00	0.84	1.16	0.00	0.00	114.62
SF	1	1	1	0	1	NA	0.47	20.35	1.00	0.00	0.00	0.00	0.88	0.00	0.00	0.05	130.62
SG	0	1	0	1	0	1	0.55	7.66	1.00	0.00	0.00	0.00	0.86	0.00	0.01	0.13	153.91
SH	0	0	0	0	0	NA	0.23	17.55	0.11	0.26	0.64	0.00	0.32	1.05	0.00	0.03	115.41
SI	0	0	0	0	0	0	0.33	7.94	0.00	0.00	0.91	0.00	0.00	0.31	0.00	0.11	165.42
SJ	0	0	0	0	0	NA	0.09	23.18	0.01	0.00	0.99	0.00	0.10	0.03	0.00	0.03	144.90
SK	0	0	0	0	0	NA	0.23	24.65	0.65	0.15	0.00	0.00	1.34	0.88	0.03	0.01	144.65
SL	0	0	0	0	0	NA	0.59	20.57	0.82	0.00	0.03	0.15	1.14	0.55	0.00	0.14	138.84
